# New frontiers in suicide vulnerability: immune system and sex hormones

**DOI:** 10.1016/j.bbih.2021.100384

**Published:** 2021-11-10

**Authors:** Giulia Lombardo

**Affiliations:** King's College London, Institute of Psychiatry, Psychology and Neuroscience, Department of Psychological Medicine, London, UK

**Keywords:** Suicide, Immune system, Sex hormones, Sex differences

## Abstract

Suicide is one of the leading causes of death worldwide and men have a higher risk of attempting and completing suicide than women. Accumulating evidence leads to a possible key role of the immune system and sex hormones in psychiatric conditions associated with suicide vulnerability (e.g., major depressive disorder). Moreover, the literature highlights a dysregulation of the immune system and altered sex hormone levels in suicidal patients. Sex hormones and the immune system may have a role in suicide risk and sex differences in suicide vulnerability. This brief review emphasises a research area focused on a possible interplay between the immune system and sex hormones that may help develop a better understanding of suicide vulnerability in the perspective of sex-specific therapeutic approaches.

## 1 Suicide vulnerability

Historically, suicide and suicide attempts were criminalised with consequent actions against the attempters (if not completed suicide), the corpse, or on the family of the departed. Nowadays, however, many countries have decriminalised suicide, possibly leading to a subsequent decrease in suicide rates (Mishara and Weisstub 2016). The World Health Organisation (WHO) counts almost 800,000 suicide deaths every year (1.4% of worldwide deaths) (Roth et al., 2018; World Health Organisation 2014). The phenomenon of suicide is multi-faceted with different grades of severity (completed suicide, suicide attempts, suicidal ideation), and can be analysed from different perspectives (e.g., cultural, psychological, and biological). Specific domains may increase the risk of suicide in childhood and adolescence: psychological factors (e.g., psychiatric disorders, previous suicide attempts, substance use), stressful life events, personality traits (e.g., neuroticism), social and educational disadvantage, family history of suicidal behaviour, family-related factors, childhood trauma, and strong belief in healing practices (Beautrais 2000; Randell et al., 2006; Vang 2013; Carballo et al., 2020). Multiple spheres are associated with suicide in adulthood: mental health, alcohol/substance abuse, recent crisis or stress, financial issues, relationship problems, or medical conditions (Logan et al., 2011). Most deaths caused by completed suicide are associated with psychiatric conditions (e.g., bipolar disorder (BD), post-traumatic stress disorder (PTSD), and schizophrenia), and 50%–66% of completed suicides are associated with affective disorders, specifically with major depressive disorder (MDD) (Arsenault-Lapierre et al., 2004; Cavanagh et al., 2003; Skogman et al., 2004). Even though the segment of the population that shows increased suicide risk is between 35 and 44 years of age (Cash and Bridge 2009); young people (<25 years of age) with MDD show a higher suicide risk than adults (Blair-West et al., 1999).

Despite suicidal ideation and behaviour being associated with the female sex (Cantor, 2000), men show higher annual suicide rates and a higher frequency of attempted suicides than women (Barrigón and Cegla-Schvartzman 2020; Oquendo et al., 2001; Skogman et al., 2004). In a National Register Based analysis (Nordic countries), male sex, previous suicide attempts, the severity of depression, and substance abuse were identified as risk factors (Isometsä 2020). Nevertheless, Skogman and colleagues highlighted sex-specific risk factors: male-specific risk factors are suicide attempts and lethal methods; female-specific risk factors are and older age and suicidal intent (Skogman et al., 2004). Men are more likely to choose violent methods and are therefore more likely to complete suicide due to the more effective methods (Stenbacka and Jokinen, 2015). On the contrary, women are more likely to choose non-violent methods (e.g., carbon monoxide poisoning) (Denning et al., 2000). The increased suicide risk in male patients may be aggravated by sex differences in the duration of the suicidal process. The process from the onset of ideation to the suicide act itself is shorter (i.e., quicker) in men than women (Neeleman et al., 2004; Van Heeringen 2001), giving less chance of timely intervention. Moreover, depressed male patients tend to show higher irritability, hyperactive behaviour, a tendency to overreact, more frequent anger attacks, and lower impulse control than depressed female patients (Winkler et al., 2005). These traits may contribute to increased difficulty in reaching an early and prompt diagnosis of depression and to subsequent increased risk of completing suicide.

The high rates of suicide worldwide, the complexity of this phenomenon, and the correlation with mental health (in particular with depression (Lombardo 2019a)) led to research investigating the underlying biological mechanisms to better understand suicide vulnerability and then, to prevent suicide.

## 2 The role of the immune system in suicide risk

The role of the immune system is detectable across various psychiatric conditions associated with suicide vulnerability (e.g., schizophrenia and PTSD) (Balhara and Verma 2012; Gradus et al., 2010; Müller et al., 2015). In particular, the immune system is one of the main biological mechanisms currently under investigation in affective disorders. Experimental activation of the immune system can induce transient depressive symptoms (Nettis et al., 2020) and a recent large study in the UK Biobank found that both male and female depressed patients have increased inflammatory levels (Pitharouli et al., 2021). In fact, depressed patients show increased pro-inflammatory biomarker levels (e.g., C-reactive protein (CRP) and tumour necrosis factor (TNF)- α) (Osimo et al., 2020). Treatment-resistant depressed (TRD) patients show even higher CRP levels (Chamberlain et al., 2019) than responders, with increased expression of immune genes (Cattaneo et al., 2020) and high rates of attempted/completed suicides (Bergfeld et al., 2018). Hence, the investigation into the role of the immune system in depression is a promising field for new target treatments and personalised medicine, with a view to reducing suicide rates. Moreover, baseline biomarker levels may affect the treatment response. For instance, research has recently begun to demonstrate that only depressed participants with higher cortisol levels at baseline assessment may benefit from cortisol synthesis inhibitors (Lombardo et al., 2019b). Furthermore, TRD patients with higher baseline inflammatory levels (high sensitivity (hs)-CRP ≥ 3 ​mg/L) may benefit from add-on treatment with minocycline, an antibiotic with anti-inflammatory and anti-depressant properties (Rosenblat and McIntyre, 2018; Nettis et al., 2021).

The immune system may also play a key role in suicide vulnerability (Brundin et al., 2017) (see Table 1). Suicidal ideation/suicide attempts are associated with increased levels of inflammatory biomarkers and stress, independently of psychiatric conditions (Enache et al., 2019) and severity of depressive symptoms (O'Donovan et al., 2013). Specifically, hs-CRP, Presumptive Stressful Life Events Scale, and the Daily Hassles and Uplifts Scale-revised independently predict suicidal risk (R2 ​= ​0.765) (Priya et al., 2016). Janelidze et al. (2011) detected higher levels of interleukin (IL)-6 and TNF-α, and lower levels of IL-2 in suicide attempters with depression than depressed patients without suicidal ideation and controls (Janelidze et al., 2011). Moreover, individuals who attempted suicide show lower GR-α mRNA and higher CRP and TNF-α mRNA than those individuals with suicidal ideation and no history of suicide attempts (and controls) (Melhem et al., 2017). In a post-mortem study focused on teenage suicide victims (age range 12–20 years old), they showed higher IL-1β, IL-6, and TNF-α mRNA levels than controls in Brodmann area 10 (Pandey et al., 2012). However, the understanding of suicide risk may be deepened by investigating those patients with low-moderate peripheral inflammation. In fact, the risk of completed suicide is quadrupled in patients with CRP >3 ​mg/L compared with patients with CRP <1 ​mg/L (Batty et al., 2016).

**Table 1:** Summary of the main findings of the discussed studies investigating immune system and suicide vulnerability.

Immune system and suicide vulnerability				
Study	Population	Sex	Relevant biomarkers	Main findings
Batty et al. (2016)	Analytical sample of 39 ​349 individuals with 26 deaths attributed to suicide. (∗Pooled data from surveys of individuals living in private households)	m, f	CRP	**CRP**: CRP >3 ​mg/L group showed 4-times the risk of completed suicide than CRP <1 ​mg/L group.
Batty et al. (2018)	The Korean Cancer Prevention Study sample of 106 643 men and 312 884 women. 1010 male suicide deaths, 1019 female suicide deaths.	m, f	White blood ​cell counts	**White blood ​cell counts**: females but not males showed a moderate association between systemic inflammation and increased suicide mortality.
Birur et al. (2017)	200 individuals: 97 controls 103 MDD patients	m, f	IL-1β, TNF-α	**IL-1β and TNF-α:** associated with suicidal ideation (and with pessimism, and lassitude) in depressed female patients.
Cabrera-Mendoza et al. (2020)	75 individuals, post-mortem: 48 suicides (38 males, 10 females); 27 controls (20 males, 7 females) Mixed diagnosis suicide group: 22 MDD 5 BD, 4 personality disorder. Control group: 2 personality disorder	m, f	Gene expression profiles	**Genes related to cell proliferation and immune response:** female-exclusive suicide genes. **Genes related to DNA binding and ribonucleic proteins:** male-exclusive suicide genes.
Janelidze et al. (2011)	47 suicidal depressed attempters, 17 non-suicidal depressed patients, 16 healthy controls.	m, f	IL-6, IL-2, TNF- α	**IL-6 and TNF-α;** higher levels in suicide attempters (vs. controls and depressed patients without suicidal ideation). **IL-2:** lower levels in suicide attempters (vs. controls and depressed patients without suicidal ideation).
Keaton et al. (2019)	66 patients with active or in remission psychiatric conditions. Mixed diagnosis group: MDD, depressive disorder not otherwise specified, dysthymic disorder, anxiety, BD-I, BD- II, BD not otherwise specified	f	IL-8	**IL-8:** negatively associated with increased suicide risk.
Köhler-Forsberg et al. (2017)	231 MDD patients	m, f	CRP	**CRP:** female patients only showed a positive association between CRP and severity of depressive, cognitive symptoms, and suicidality
Melhem et al. (2017)	38 suicide attempters, 40 patients with suicidal ideation with no prior history of attempts, 37 healthy controls Mixed diagnosis group: MDD, BD, psychotic disorder, anxiety, PTSD, ADHD, Eating disorders, Alcohol dependence, personality disorders.	m, f	GR- α mRNA, CRP mRNA, TNF- α mRNA	**GR- α mRNA** lower in suicide attempters (vs. controls and vs. individuals with suicidal ideation and past suicide attempts). **CRP and TNF- α mRNA** higher in suicide attempters (vs. controls and vs. individuals with suicidal ideation and past suicide attempts).
Pandey et al. (2012)	24 teenage suicide victims, 24 teenage controls (post-mortem) Mixed diagnosis group: MDD, PTSD, alcohol and substance abuse, ADHD, adjustment disorder, conduct disorders, borderline personality disorder, PTSD, dissociative disorder.	m, f	IL-1β mRNA, IL-6 mRNA, and TNF-α mRNA	**IL-1β, IL-6, and TNF-α mRNA** higher in suicide victims vs. controls in Brodmann area 10.
Tonelli et al. (2008)	34 suicide victims, 17 controls (post-mortem). Available psychological information on 12 suicide victims: 9 MDD, 1 BD-I, 2 alcohol abuse	m, f	IL-13 mRNA, IL-4 mRNA, IL-3 mRNA	**IL-13 mRNA** expression increased in male suicide victims (vs. controls) in Brodmann area 11. **IL-4 and IL-3 mRNA** expression increased in female suicide victims (vs. controls) in Brodmann area 11.
**SUICIDE METHOD-SPECIFIC INFLAMMATORY BIOMARKERS**				
**Study**	**Population**	**Sex**	**Relevant biomarkers**	**Main findings**
Isung et al. (2014)	Plasma: 58 suicide attempters (23 men, 35 women) CSF: 43 suicide attempters (15 men, 28 women) Mixed group diagnosis: MDD, personality disorder, alcohol abuse.	m, f	IL-6	**IL-6 (plasma)** higher in violent methods suicide attempters vs. non-violent method suicide attempters. **IL-6 (plasma):** showed a significant positive correlation with impulsivity. (∗*IL-6 (CSF): no statistically significant findings linked to violence of suicide method and impulsivity*).
Lindqvist et al. (2009)	63 suicide attempters, 47 healthy controls Mixed diagnosis group (suicide attempters): MDD, depression not otherwise specified, adjustment disorder, substance abuse disorder, dysthymia, psychosis, eating disorder (or no diagnosis)	m, f	IL-6	**IL-6 (CSF)** higher suicide attempters vs. controls; and higher in violent method suicidal attempters than non-violent method suicide attempters.
Noroozi et al., 2018	229 suicidal attempters with soft suicide methods, 235 suicide victims, 290 healthy controls.	m, f	IL-8 polymorphism A T A haplotype	**IL-8 polymorphism A T A haplotype** (rs4073, rs2227306 and rs1126647) higher frequencies in violent method suicidal individuals than non-violent method suicidal individuals.

Note: ADHD (Attention-deficit/hyperactivity disorder); BD (bipolar depression); CRP (C-reactive protein); CSF (cerebrospinal fluid); f (female); IL (interleukin); m (male); MDD (major depressive disorder); PTSD (Post-traumatic stress disorder); TNF (tumor necrosis factor).

Interestingly, the literature highlights sex differences in the biochemical mechanisms associated with this phenomenon. Previous studies investigating inflammatory biomarkers in suicide risk have reported inconsistent findings in analysing the activation of the immune system in male and female patients.

Women but not men may show an association between systemic inflammation and increased suicide risk. For instance, females with higher white blood cell counts (≥5.9x109 ​cells/L) have a 30% increased risk of death by suicide (Batty et al., 2018). Moreover, depressed female patients show a positive but low correlation between suicidal thoughts and TNF-α (r=0.33 p=0.03) (Birur et al., 2017). Additionally, Köhler-Forsberg and colleagues detected a positive association between CRP levels, severity of depressive/cognitive symptoms, and suicidality in women but not in men (suicidality: coefficient of increase for each standard deviation of LnCRP =0.12 p=0.05) (Köhler-Forsberg et al., 2017).

Interestingly, there may be a need to further investigate sex-specific biological and inflammatory profiles in suicidal patients. A recent study by Cabrera-Mendoza and colleagues detected sex-specific gene expression in suicidal patients: cell proliferation and immune response in women, and DNA binding and ribonucleic proteins in men (Cabrera-Mendoza et al., 2020). Tonelli et al. (2008) identified sex-specific markers in a post-mortem study investigating the mRNA cytokine expression in the prefrontal cortex. The authors detected increased IL-13 mRNA expression in male suicide victims and increased IL-4 and IL-3 mRNA expression in female suicide victims (in comparison with controls) (Tonelli et al., 2008). Moreover, in a cohort of female patients with mood and anxiety disorders, IL-8 was inversely associated with increased suicide risk in the ridge regression model (β=−0.03±0.02, p<0.05) (Keaton et al., 2019).

Furthermore, as mentioned earlier, males have a higher tendency to choose violent suicide methods compared with females. Lindqvist and colleagues detected higher levels of CSF IL-6 in violent-method suicide attempters than non-violent method suicide attempters (Lindqvist et al., 2009). Moreover, impulsivity and aggression are linked to completed suicide in males (Mann et al., 2005). Interestingly, increased levels of IL-6 are associated not only with violent suicide attempt methods but also with impulsivity (r=0.39 p< 0.01) (Isung et al., 2014). Additionally, Noorozi and colleagues (2018) detected higher frequencies of A T A haplotype (rs4073, rs2227306, and rs1126647 respectively) of IL-8 polymorphisms in suicidal individuals who endeavoured hard methods than suicidal individuals who endeavoured soft methods (Noroozi et al., 2018).

This evidence highlights the need to detect sex-specific markers associated with suicide risk.

The aforementioned altered levels of and sex differences in inflammatory biomarkers in suicidal patients may be linked to the immunomodulator proprieties of gonadal hormones and a possible role of sex hormones in suicide vulnerability.

## 3 The role of sex hormones in suicide risk

Sex hormones and their fluctuations affect the neurobiology of affective disorders (Rubinow and Schmidt 2019), the sex differences in susceptibility to stress, and the increase in inflammatory levels (Slavich and Sacher 2019). Moreover, the literature highlights altered levels of gonadal hormones in both male and female suicidal patients (see Table 2). Therefore, the investigation of sex hormones may be a key factor in understanding sex differences and the inflammatory profile in suicidal patients.

**Table 2:** *Summary of the main findings of the discussed studies investigating sex hormones and suicide vulnerability*.

Sex hormones and suicide vulnerability				
Study	Population	Sex	Relevant biomarkers	Main findings
Baca-Garcia et al. (2010)	431 suicide attempters Mixed group diagnosis: 179 with history of psychiatric disorders; 60 mood disorder; 25 substance use disorders.	f	Estradiol, progesterone	**Estradiol, progesterone:** low estradiol/low progesterone states (menstrual phase, amenorrhea and menopause) associated with attempted suicides.
Behera et al. (2019)	547 subject autopsies: 282 suicidal deaths; 265 non-suicidal deaths.	f	Menstrual cycle phases, corpus luteum	**Secretory/luteal phase**: 56.9% of suicides **Menses and luteal phase**: increased risk of completed suicide **Corpus luteum in the right ovary**: 43 suicidal victims and 14 non-suicidal deaths. **Corpus luteum in the left ovary**: 3 suicidal victims and 11 non-suicidal deaths.
Mousavi et al. (2014)	111 suicide attempters (62.2% single attempter; 37.8% recurrent attempters)	f	Estrogen, progesterone	**Luteal phase:** higher percentage of suicide attempts (vs. follicular phase) **Progesterone** higher in recurrent attempters (≥3 suicide attempts) vs. ​< ​3 suicide attempts. **Estrogen:** no association with suicidal attempt rankings (first attempters, second attempters, three or more attempters)
Sher et al. (2012)	67 BD patients with at least one past suicide attempt Mixed group diagnosis: BD-I and BD-II	m, f	Testosterone	**Testosterone**: male patients showed higher testosterone levels, lethality of suicide attempts and of depressive episodes than female patients, with testosterone being associated with manic episodes and number of suicide attempts.
Sher et al. (2014)	51 BD patients in a depressive or mixed episode (with at least one past suicide attempt)	f	Testosterone	**Testosterone**: higher baseline levels positively associated with future suicide attempts (increase by 10 ​ng/dl increases probability of suicide attempt 16.9 times).
Stefansson et al. (2016)	47 subjects: 28 suicide attempters; 19 healthy controls. Mixed group diagnosis: affective disorders, adjustment and anxiety disorders, substance-related disorder, personality disorder (1 patient organic personality syndrome diagnosis)	m, f	Testosterone	**Testosterone:** higher testosterone (CSF and plasma) in male suicide attempters vs. male controls. CSF testosterone/cortisol ratio showed a significant positive correlation with both impulsivity and aggressiveness. **Testosterone:** no significant differences in testosterone (CSF and plasma) between female suicide attempters vs. female controls.
Tripodianakis et al. (2007)	136 subjects: 80 suicide attempters; 56 controls Mixed group diagnosis: adjustment disorder, personality disorder, MDD, schizophrenia	m	Testosterone	**Testosterone**: lower testosterone levels in attempters vs. controls. **LH**: marginally higher LH levels in attempters vs. controls **Testosterone and suicide method**: violent suicide method attempters have lower testosterone levels than non-violent suicide method attempters.
Zhang et al. (2015)	490 subjects: 245 suicide attempters; 245 controls Mixed group diagnosis: depression, anxiety, dysfunctional impulsivity and mental disorder not otherwise specified.	m, f	Testosterone	**Testosterone**: higher testosterone in suicide attempters vs. controls

Note: BD (bipolar depression); CSF (cerebrospinal fluid); f (female); LH (luteinizing hormone); m (male); MDD (major depressive disorder).

In women, suicide attempts are linked to decreased levels of estrogen (Sublette 2020), and increased levels of testosterone (p=0.017) (Zhang et al., 2015). This is detectable also in bipolar depression. BD female patients (depressive episode or mixed episode) show a positive association between testosterone levels and the number of suicide attempts (r=0.408 p<0.01) (Sher et al., 2014). Moreover, the menstrual cycle may be a significant component in suicide risk (Leenaars et al., 2009). For instance, Behera et al. (2019) reported an increased risk of completed suicide among women during their menses and luteal phase (Behera et al., 2019). Additionally, patients with a number equal to or greater than 3 suicide attempts have higher levels of progesterone and a higher percentage of suicide attempts in the luteal phase than the follicular phase (Mousavi et al., 2014). On the contrary, Baca-Garcia and colleagues observed that female life phases characterised by low levels of progesterone (and estradiol), such as menopause, were associated with female attempted suicides (Baca-Garcia et al., 2010). In our recent systematic review focused on the interplay of sex hormones and the immune system in affective disorders, we suggest a protective role of exogenous female sex hormones in sub-groups characterised by estrogen deprivation (Lombardo et al., 2021).

In men, completed suicide rates and suicide attempts are linked to increased androgen levels (Sublette 2020; Zhang et al., 2015), and high androgen levels are associated with aggressive behaviour (Pompili et al., 2013). Interestingly, aggressiveness (and impulsivity) shows a positive and moderate association with testosterone/cortisol ratio (cerebrospinal fluid) in male suicide attempters (r=0.67 p=0.02) (Stefansson et al., 2016). In a cohort of BD patients (mixed group of bipolar depression I and bipolar depression II), male patients showed positive but low correlations between testosterone and manic episodes (r=0.32 p=0.02) and between testosterone and the number of suicide attempts (r=0.35 p<0.01) (Sher et al., 2012). On the contrary, a mixed diagnosis male suicide attempter group (adjustment disorder, personality disorder, MDD, schizophrenia) showed lower testosterone levels than controls; with a significant difference in schizophrenic patients and a statistical trend in MDD patients (both diagnosis groups revealed lower testosterone levels than controls). Nevertheless, violent-method suicide attempters showed lower testosterone levels than non-violent method suicide attempters (Tripodianakis et al., 2007).

Several studies detect an immune-modulating role of gonadal hormones, as we extensively discuss in our recent aforementioned review (Lombardo et al., 2021). Specifically, estrogen can have both anti-inflammatory and pro-inflammatory properties, depending on various aspects, such as the target organ or concentration (high vs. low) (Straub 2007). Testosterone is known to have mainly anti-inflammatory properties (e.g., by inhibiting TNF-α, IL-6, IL-1 expression). For instance, low testosterone levels are associated with pro-inflammatory biomarker expression (Bianchi 2019; Bobjer et al., 2013). Specifically, testosterone replacement administration in men with androgen deficiency reduced pro-inflammatory cytokines (i.e., TNF-α and IL-1β), and increased anti-inflammatory cytokines (i.e., IL-10) (and cholesterol) (Malkin et al., 2004). However, there is still a need for research measuring androgen in females and estrogen measure analyses in males, and for the selection of patients with moderate-high inflammatory levels at the baseline for a better understanding of suicide vulnerability. In fact, the literature also highlights a pro-inflammatory effect of testosterone in women and a pro-inflammatory effect of estrogen in men. Testosterone (along with androstenedione and dehydroepiandrosterone sulphate) shows a positive correlation with white blood cell count and it is a predictor of leukocyte count in women with polycystic ovary syndrome (PCOS) (Rudnicka et al., 2020). In men with rheumatoid arthritis, estradiol shows a positive and strong correlation with inflammation (Tengstrand et al., 2003). Specifically, the balance between estradiol and testosterone may be relevant in modulating the immune system. In fact, van Koeverden and colleagues detected a negative correlation between low testosterone/estradiol ratio and systemic inflammation in men with atherosclerotic disease (Van Koeverden et al., 2019).

All this evidence highlights possible future directions in exploring new method-specific and sex-specific biomarkers in suicidal patients.

## 4 Challenges for future research

There is a lack of studies in this area which makes it difficult to reach a clear understanding of the interplay between sex hormones and the immune system in suicidal patients. Moreover, the reported information is association data, and the strength of these associations is low-moderate. Thus, it is necessary to further evaluate the strength of these associations, for example by following the Bradford Hill criteria (Hill 1965; Fedak et al., 2015), using systematic review/meta-analysis formats and a systematic approach. Most studies did not stratify, nor select, the patients based on inflammatory biomarker levels. In fact, selecting suicidal patients who attempted suicide or who show suicidal intent, may help in clarifying the role of altered inflammatory biomarker and sex hormone levels (and their interplay) in this population. Furthermore, the inconsistency across the literature investigating inflammatory biomarker levels in suicide vulnerability needs further investigation. Moreover, the plethora of factors involved in this phenomenon requires an investigation of, and comparison between, the role of biological and non-biological (sociocultural, religious, and behavioural) factors, as well as sex differences, involved in suicide vulnerability.

## 5 Conclusion

The literature highlights that altered sex hormone levels correlate with increased suicide risk (e.g., decreased estrogen levels in female patients correlate with suicide attempts and increased androgen levels may play a role in higher rates of completed suicides in men). Furthermore, several studies reported altered immune system activation in suicide attempters/victims, also with differences in inflammatory profiles between suicide methods (i.e., violent vs. non-violent). However, even though several studies described the immune modulator role of gonadal hormones, there are no studies in the literature to our knowledge that specifically investigate the interplay between the two factors of interest (i.e., sex hormones and immune system) in suicide risk and sex differences in suicide vulnerability.

**Fig. 1:**
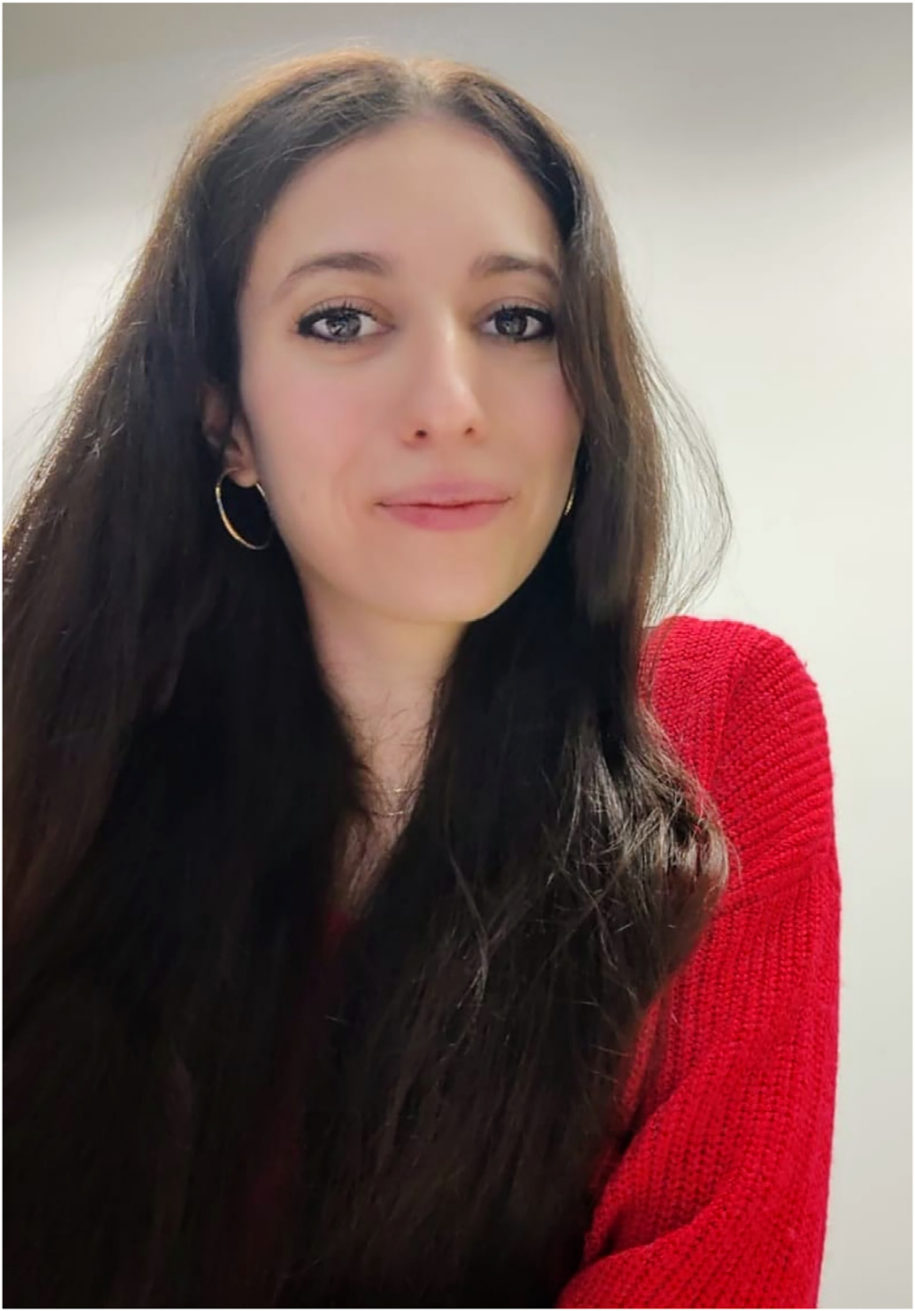
**Giulia Lombardo** Giulia Lombardo is a full-time PhD student in the Stress, Psychiatry and Immunology (SPI)-Lab, and in the Psychoimmunometabolix and Interaction with the Environment (PIXIE)-Lab at the Department of Psychological Medicine, Institute of Psychiatry, Psychology and Neuroscience (IoPPN), King's College London. While completing her Master of Psychology at the University of Turin ​(Italy) "Body and Mind Sciences" course, with a particular interest in the dialogue between biological and psychological aspects in mental health, she worked as a research assistant ​at the King's ​College London to ​follow her passion in the interplay between mind and body. She believes that the study of biological dynamics in psychiatric illnesses will help in creating a sex-specific medicine and tailored treatments in order to offer the best rehabilitation pathway for those patients in need. Therefore, after the Master, Giulia decided to pursuit her passion in her PhD project investigating the (Barrigon and Cegla-Schvartzman, 2020) role of inflammation and sex hormones in depression and in sex differences in treatment response, with a particular focus on major depressive disorder and treatment resistant depression. Giulia is also one of the main academic leads of the Immunopsychiatry Meetings organised by the PIXIELab, which represent an opportunity for discussion and debate with the aim to create ​an international network of ​Immunopsychiatry.

Given the altered levels of inflammation and sex hormones in suicidal patients (and in psychiatric conditions associated with suicide risk), lack of consideration for the interaction between the immune system and gonadal hormones can limit the progress in knowledge of suicide vulnerability and can preclude new preventive tailored therapeutic strategies (see Fig. 1).

## Declaration of competing interest

The author declares that there is no conflict of interests.
